# A Myeloid Signature-Based Nomogram Predicts the Postoperative Recurrence of Intrahepatic Cholangiocarcinoma

**DOI:** 10.3389/fmolb.2021.742953

**Published:** 2021-10-13

**Authors:** Jing Liang, Hui Zhou, Xiang-Qi Huang, Yan-Fei Liu, Lei Zhang, Dan He, Yongmei Cui, Jinrui Guo, Kunpeng Hu, Chong Wu

**Affiliations:** ^1^ Department of Pathology, The Third Affiliated Hospital, Sun Yat-sen University, Guangzhou, China; ^2^ Department of General Surgery, The Third Affiliated Hospital, Sun Yat-sen University, Guangzhou, China; ^3^ Department of Gastrointestinal Surgery, The Seventh Affiliated Hospital, Sun Yat-sen University, Shenzhen, China; ^4^ Department of Obstetrics and Gynecology, The Third Affiliated Hospital, Sun Yat-Sen University, Guangzhou, China; ^5^ Department of Biliary-Pancreatic Surgery, The Third Affiliated Hospital, Sun Yat-sen University, Guangzhou, China; ^6^ MOE Key Laboratory of Gene Function and Regulation, State Key Laboratory of Biocontrol, School of Life Sciences, Sun Yat-sen University, Guangzhou, China

**Keywords:** CD11b, CD169, nomogram, intrahepatic cholangiocarcinoma, recurrence

## Abstract

Intrahepatic cholangiocarcinoma (iCCA) is the second most common cancer in liver, with a high recurrence rate after surgery. Recently, we identified a CD11b-CD169-based myeloid response score (MRS), which showed remarkable prognostic potential in hepatocellular carcinoma (HCC). Here, we aimed to verify the prognostic value of the MRS in iCCA and establish an MRS-based nomogram to predict the postoperative prognosis of iCCA patients. From April 2005 to March 2017, a total of 84 patients from the Third Affiliated Hospital of Sun Yat-sen University were enrolled. Preoperative clinical information and surgical specimens of enrolled patients were collected. Among these, tissues from 75 patients passed the clinical data quality control and the staining quality control. The protein expression of CD11b and CD169 in iCCA samples were detected by immunohistochemistry (IHC). Kaplan-Meier analysis and receiver operating characteristic (ROC) curves revealed that the MRS had a high discriminatory ability for predicting the time to recurrence (TTR) of iCCA patients after surgery. Three independent risk factors selected by a Cox proportional hazards regression analysis, namely, the MRS, the tumor size and the status of vascular invasion, were included to construct a nomogram to predict the recurrence of iCCA after resection surgery. ROC curves, calibration analysis and decision curve analysis (DCA) suggested that this nomogram had notable discriminatory power, stability and clinical usefulness in predicting the postoperative recurrence. Together, we explored the prognostic value of the MRS in iCCA, and constructed an MRS-based nomogram which may help to predict postoperative recurrence and aid clinical decisions for iCCA patients.

## Introduction

Intrahepatic cholangiocarcinoma (iCCA), ranked as the second most common primary liver cancer, is a highly aggressive and lethal hepatobiliary neoplasm (Bertuccio et al., 2019). Currently, only a part (30%) of patients are eligible for curative surgery (Sirica et al., 2019; Banales et al., 2020) and the high postoperative recurrence rate (40–80%) contribute primarily to the dismal prognosis of iCCA (Mavros et al., 2014; Wirth and Vogel 2016). Therefore, it is essential to identify patients with high risk of recurrence after hepatic resection for individualized postoperative management.

Myeloid cells, including macrophages, dendritic cells, granulocytes and myeloid-derived suppressor cells, are a group of heterogeneous innate immune cells that arise from the common myeloid precursor cells. These cells construct a dynamic and interactive local inflammatory milieu and exert distinct or even opposing influences on the tumor development (Engblom et al., 2016). CD11b (also known as integrin αM), a common myeloid marker which widely expressed on myeloid-derived suppressor cells, neutrophils, and macrophages, plays an important role in cell adhesion, migration, chemotaxis, phagocytosis, and respiratory burst activity (Arnaout 1990; Schmid et al., 2018; Clement et al., 2019). An increasing number of researches have revealed the prognostic value and the therapeutic potential of CD11b. For example, in pancreatic cancer, targeting CD11b could attenuate the immunosuppressive myeloid response and overcome resistance to immunotherapy (Panni et al., 2019). CD169 (also known as Siglec-1 or sialo adhesin) is usually identified as a macrophage marker in most tumors. CD169^+^ sinus macrophages in the lymph nodes are correlated with a favorable prognosis in patients with bladder cancer, endometrial carcinoma, colorectal carcinoma and prostate cancer (Ohnishi et al., 2013; Ohnishi et al., 2016; Stromvall et al., 2017; Asano et al., 2018). We have shown that the abundance of CD169^+^ tumor-associated macrophages is correlated with good patient outcomes in hepatocellular carcinoma (HCC) and gastric cancer (Zhang Y. et al., 2016; Li et al., 2017). In addition, this protein could also be detected on lymph node CD8^+^ T cells in colon cancer, possibly due to their close interaction with sinus macrophages. The presence of CD169^+^CD8^+^ T cells in the lymph node is also associated with a favorable prognosis (Zhang J. et al., 2016). In contrast, the higher expression level of CD169 mRNA in breast cancer is associated with a shorter disease-specific and recurrence-free survival (Cassetta et al., 2019). These studies further underpinned the hypothesis that phenotypes/functions of myeloid cells are organ and cancer specific. However, in iCCA, the *in situ* expression and the prognostic value of CD11b and CD169 remains unclear.

Our understanding of the innate immune microenvironment in tumor has been substantially deepened in the past decade, but the clinical transformation of this information still remains a challenge (Galon and Bruni 2020). Recently, nomograms have been developed in many cancers to assess individual probability of a clinical event such as cancer recurrence or death and provides a unique opportunity to improve individual risk assessment compared with conventional staging systems (International Bladder Cancer Nomogram Consortium et al., 2006; Karakiewicz et al., 2007; Hasita et al., 2010). Yet, in iCCA, most of the established nomograms have been developed on the basis of clinicopathological characteristics only. Few immune or myeloid markers have been tested for their prognostic potential and incorporated into a nomogram to contribute to a better prognostic and predictive algorithm.

In our previous study, we identified a simple CD11b-CD169-based myeloid response score (MRS) by screening nine myeloid markers which covered the main tumor-infiltrating myeloid subtypes, and constructed MRS-based predictive nomograms to predict the prognosis of postoperative HCC patients (Wu et al., 2020). Here, we evaluated the CD11b and CD169 expression in iCCA specimens through *in situ* immunohistochemistry (IHC) and validated the prognostic potential of CD11b-CD169-based MRS in iCCA. Importantly, we further constructed an MRS-based nomogram exhibiting remarkable discriminatory ability, accuracy, and clinical usefulness in predicting the postoperative recurrence of iCCA.

## Materials and Methods

### Patient Information

Formalin-fixed, paraffin-embedded tumor tissues were collected from 84 patients who underwent iCCA resection at the Third Affiliated Hospitals of Sun Yat-Sen University (from April 1st, 2005 to March 3rd, 2017). Among these, tissues from 75 patients passed the clinical data quality control and the staining quality control. The inclusion criteria were as follows: pathologically confirmed iCCA; no preoperative anticancer therapies; no diagnosis or history of any other concurrent malignancies; no concurrent autoimmune diseases, HIV or syphilis; and available follow-up data. After surgery, all patients were regularly followed. Serum tumor markers, abdominal ultrasonography and chest radiography were performed for surveillance for recurrence. Further examinations, including CT, hepatic angiography, and biopsies (when necessary), were performed when tumor recurrence or metastasis was suspected. All iCCA patients were staged according to the 8th edition of the American Joint Committee on *Cancer* (AJCC) staging system. The median follow-up was 31 months, ranging from 0 to 131 months. This study was approved by the Ethic committee of the participating hospital. Written informed consents which were accordance with the Declaration of Helsinki were signed by all patients. The clinicopathological characteristics of the patients enrolled in this study were summarized in Table 1.

**TABLE 1:** Clinical characteristics of patients with iCCA.

Variable	No. of patients	%
No. of patients	75	100
Age; median(range), yr	56.1 (23–78)	
Gender		
Male	40	53.3
Female	35	46.7
Tumor size; median (range), cm	6.35 (1.4–18.1)	
Tumor location		
Perihilar	9	12.0
Peripheral	66	88.0
Histological subtypes		
Papillary adenocarcinoma	5	6.7
Tubular adenocarcinoma	47	62.7
Others	23	30.7
Differentiation		
Well + Moderate	54	72.0
Poor	21	28.0
Perineural invasion		
Positive	29	38.7
Negative	46	61.3
Vascular invasion		
Positive	51	68.0
Negative	24	32.0
T stage		
T1	20	26.7
T2	38	50.7
T3	15	20.0
T4	2	2.7
*N* stage		
N0	28	37.3
N1	47	62.7
Distant metastasis		
Negative	65	86.7
Positive	10	13.3
TNM stage		
I	10	13.3
II	14	18.7
III	41	54.7
IV	10	13.3

### IHC and Evaluation of IHC Features

The paraffin-embedded samples were cut into 4 μm thickness sections used to immunohistochemical analysis as previously described (Wu et al., 2020). Briefly, the tissue sections were placed in xylene for dewaxing, and immersed in a decreasing concentration of ethanol for rehydrating, reacted with 0.3% H_2_O_2_ for 10 min at room temperature for endogenous peroxidase eliminating and subsequently subjected to heat-mediated retrieval in citrate buffer (pH = 6.0) for antigen retrieval. Then slides were incubated with primary CD11b antibodies (1:2000, EPR1344, Abcam, United Kingdom) and primary CD169 antibodies (1:200, NS0, R&D Systems, United States) overnight at 4°C, followed by incubation with a horseradish peroxidase-conjugated secondary antibody at 37°C for 30 min. Finally, immunostaining was visualized with diaminobenzidine (DAB, K5007, Dako, United States) and brown color indicated positive staining.

The digital whole image of the IHC stained slides were scanned at 20× magnification by an automatic digital slide scanner Pannoramic MIDI (3DHISTECH, Hungary). Then five tumor microscopic fields were randomly selected using 3DHISTECH Panoramic Viewer. The number of CD11b positive or CD169 positive cells was manually counted independently by pathologists who were blinded to the patients’ clinical characteristics and outcomes. The tissue staining results were independently analyzed by two pathologists to reduce bias and the mean of the counting results were used. Concurrent analysis using a double-headed microscope was conducted if significant discrepancies existed in the results obtained by these two pathologists. There was a consensus regarding the final results of all samples.

### Calculation of Myeloid Response Score (MRS)

The MRS was calculated as previously described: MRS = 0.161 × CD11b − 0.106 × CD169 + 35 (0 ≤ MRS ≤ 100) (Wu et al., 2020). The optimal cutoff values of MRS were generated by X-tile program (Camp et al., 2004) to stratify iCCA patients into subgroups (Supplementary Figure S1).

### Construction and Validation of MRS-Based Nomogram

A nomogram was constructed to predict the 1, 2 and 3 years recurrence of iCCA after resection surgery by including all independent prognostic factors on multivariable analyses using the *rms* package in R software version 4.0.0 (http://www.r-project.org/). Time-dependent receiver operating characteristic (ROC) curve analysis was established for examining the performance of the nomogram model using the R package *survivalROC*. Area under curve (AUC) of time-dependent ROC curves were estimated with 500× bootstrap resampling for each parameter. Decision curve analysis (DCA) was conducted to assess whether nomogram-assisted decisions could improve patients’ clinical outcomes by quantifying the net benefits at different threshold probabilities (i.e., the probability that triggers a medical intervention, equating to the probability at which the harm of a false-positive intervention exceeds the harm of a false-negative non-intervention).

### Statistical Analysis

Statistical tests were performed with GraphPad Prism 5 (GraphPad Software, United States), IBM SPSS Statistics 21.0 (IBM, United States) or R software (version 4.0.0). Time to recurrence (TTR) was defined as the time from resection to recurrence or from resection to the last observation for patients without recurrence. Overall survival (OS) was defined as the time from resection to death or from resection to the last observation for surviving patients. Stratifying patients into subgroups based on the optimal cutoffs generated by X-tile plots. Kaplan–Meier estimates were calculated and compared using the log-rank test. Univariate and multivariate analyses were performed using the Cox proportional hazards model. Significant risk factors identified from the univariate analysis were analyzed in a multivariate Cox proportional hazards model along for each potential risk factor. The association between variables was evaluated using the Chi-Square (*χ* ^2^) test. A *p*-value of less than 0.05 was considered to indicate statistical significance.

## Results

### Clinicopathologic Characteristics

A total of 75 eligible patients with iCCA from April 2005 to March 2017 were included in the study. Table 1 lists the baseline clinicopathological features of enrolled patients. Patients’ median age at diagnosis was 56.1 years (range, 23–78 years). Mean tumor diameter was 6.35 cm (range, 1.4–18.1 cm). Tumors were classified as well or moderate differentiation in 54 (72.0%), and poor differentiation in 21 (28.0%). A total of 29 patients (38.7%) had perineural metastasis, 51 patients (68.0%) had vascular invasion and 10 patients (13.3%) had distant metastasis.

**FIGURE 1:**
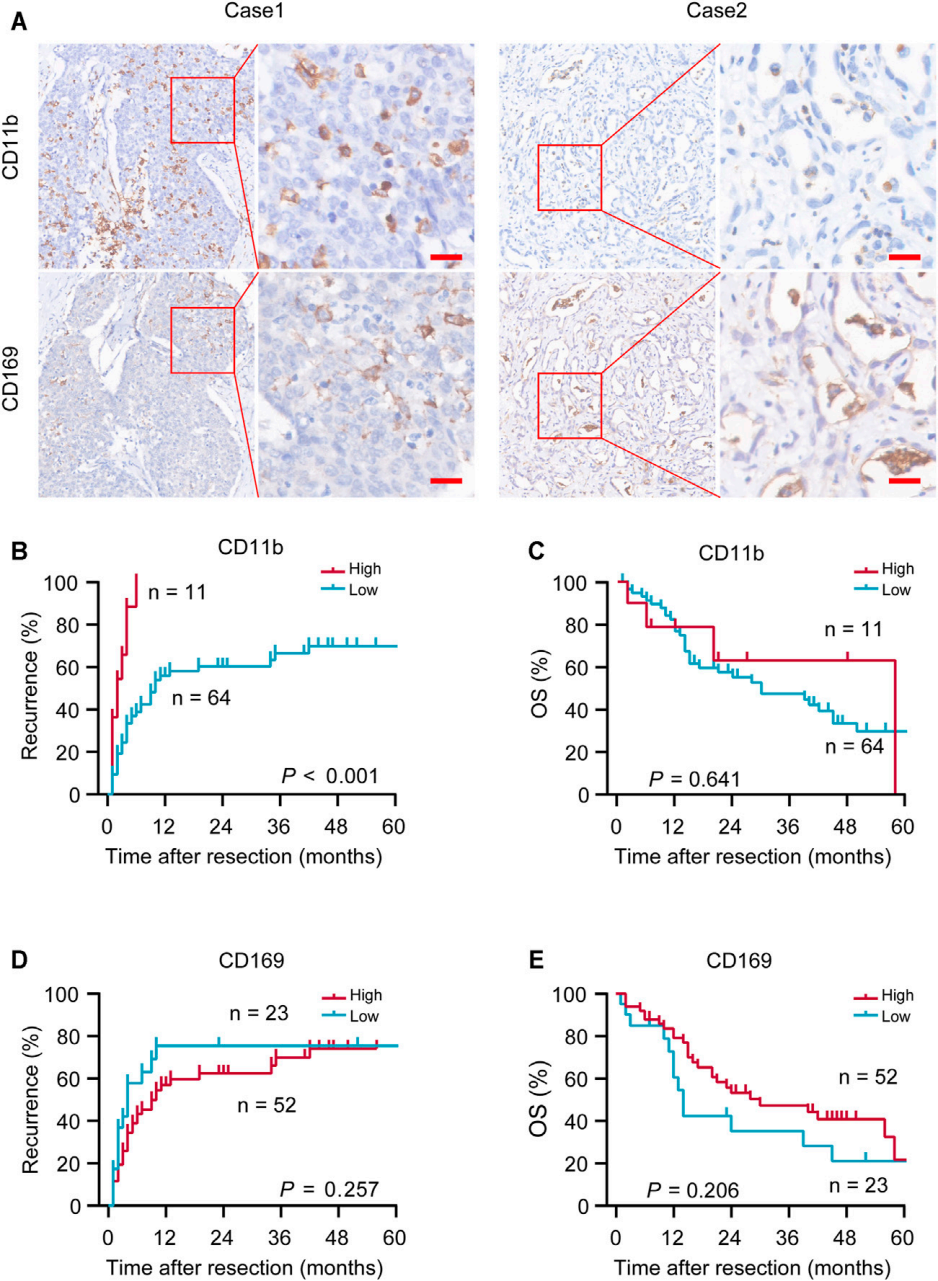
Representative immunohistochemical (IHC) images and prognostic values of CD11b and CD169. **(A)** Representative *in situ* IHC images of CD11b and CD169 in intrahepatic cholangiocarcinoma (iCCA) after surgery. **(B–E)** Kaplan–Meier survival estimates of the recurrence and overall survival (OS) were performed according to the expression of CD11b and CD169 in iCCA. Scale bar, 20 μm; *p* values were calculated by log-rank.

CD11b and CD169 were mainly presented on cell membranes in iCCA tumor tissues (Figure 1A). Optimal cutoff values were calculated using the X-tile program and the patients were stratified into two subgroups (CD11b^High^ and CD11b^Low^; CD169^High^ and CD169^Low^). Patients of CD11b^High^ group had greater possibility of recurrence than patients of CD11b^Low^ group (log-rank, *p* < 0.001; Figure 1B), while it had no significant correlation with the OS of patients with iCCA (log-rank, *p* = 0.641; Figure 1C). CD169 had no significant predictive value for TTR (log-rank, *p* = 0.257) or OS (log-rank, *p* = 0.206) (Figures 1D,E). These results suggested that CD11b or CD169 alone had limited prognostic performance in iCCA.

### The Association Between the MRS and Postoperative Recurrence

**FIGURE 2:**
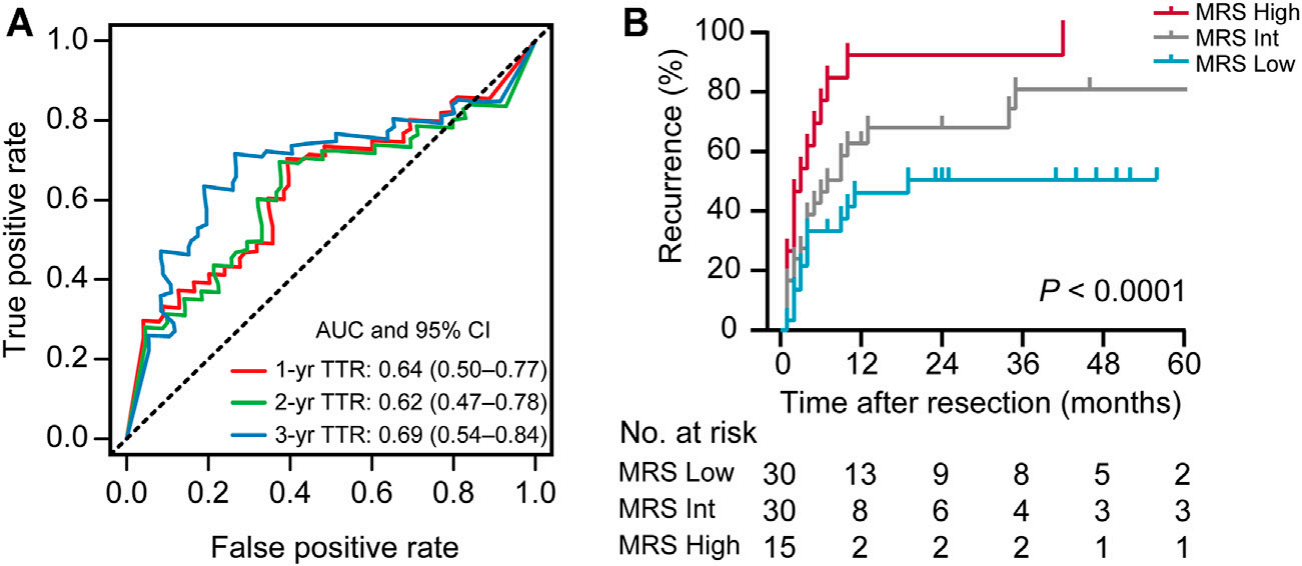
The prognostic performance of the MRS in iCCA. **(A)** Receiver operating characteristic (ROC) curve analysis showed the discriminatory ability of MRS (as a continuous score) for predicting 1, 2, and 3 years recurrence of iCCA after resection. **(B)** Kaplan-Meier survival analysis of time to recurrence (TTR) of patients in iCCA according to the MRS (as a categorical variable). Cutoffs were determined using the X-tile program as described in Supplementary Figure S1, dividing the cohort into three subgroups (MRS^Low^, 0–35.7; MRS^Int^, 35.7–97.0; MRS^High^, 97.0–100; Supplementary Figure S1). The area under curve (AUC) was shown as the mean and 95%CI.

Recently, we defined a simple and reliable prognostic signature in HCC, called the myeloid response score (MRS) (Wu, et al., 2020). To evaluate the potential role of MRS in human iCCA, we first calculated MRS as previously described: MRS = 0.161 × CD11b − 0.106 × CD169 + 35 (0 ≤ MRS ≤ 100). ROC curve analysis revealed that the MRS had a moderate discriminatory ability for predicting the TTR of patients after iCCA resection (Figure 2A), which was supported by the Kaplan-Meier curves and log-rank test (*p* < 0.0001,Figure 2B). The calculated AUC was 0.64 for 1-year recurrence, 0.62 for 2-years recurrence and 0.69 for 3-years recurrence (Figure 2A). Chi-square test showed that tumor differentiation was associated with MRS (*p* = 0.007, Table 2). Univariate and multivariate analysis indicated that the MRS [as a continuous score, hazard ratio (HR), 1.012; 95% CI, 1.003–1.020; *p* = 0.007], along with the tumor size (HR, 3.413; 95% CI, 1.116–5.365; *p* = 0.001) and the vascular invasion status (HR, 3.315; 95% CI, 1.534–7.165; *p* = 0.002) were independent risk factors for recurrence of iCCA (Table 3). However, MRS was not associated with the OS of iCCA patients (HR, 1.001; 95% CI, 0.991–1.012; Supplementary Table S1). These results suggest that the MRS may help to predict the postoperative recurrence of iCCA.

**TABLE 2:** Correlation between the MRS Grouping and clinicopathological parameters.

Variable	MRS^Low^ (*n* = 30)		MRS^Int^ (*n* = 30)*p* value		MRS^High^ (*n* = 15)		*p* value
	No	%	No	%	No	%	
Age (years)							0.498^a^
Median	58.5		59		56		
Range	37–70		25–78		23–69		
Gender							
Male	17	56.7	14	46.7	9	60	0.626
Female	13	43.3	16	53.3	6	40	
Tumor size							
<5 cm	10	33.3	13	43.3	3	20	0.295
≥5 cm	20	66.7	17		12	80	
Tumor location							
Perihilar	3	10	5	16.7	1	6.7	0.567
Peripheral	27	90	25	83.3	14	93.3	
Histological subtypes							
Papillary adenocarcinoma	3	10	1	3.3	1	6.7	0.185
Tubular adenocarcinoma	21	70	20	66.7	6	40	
Others	6	20	9	30	8	53.3	
Differentiation							
Well + Moderate	23	76.7	25	83.3	6	40.0	0.007
Poor	7	23.3	5	16.7	9	60	
Perineural invasion							
Positive	14	46.7	8	26.7	7	46.7	0.219
Negative	16	53.3	22	73.3	8	53.3	
Vascular invasion							
Positive	19	63.3	19	63.3	13	86.7	0.223
Negative	11	36.7	11	36.7	2	13.3	
T stage							
T1 + T2	23	76.7	24	80	11	73.3	0.875
T3 + T4	7	23.3	6	20	4	26.7	
*N* stage							
N0	11	36.7	11	36.7	6	40	0.972
N1	19	63.3	19	63.3	9	60	
Distant metastasis							
Negative	26	86.7	24	80	15	100	0.177
Positive	4	13.3	6	20	0	0	
TNM stage							
I + II	9	30	10	33.3	5	33.3	0.955
III + IV	21	70	20	66.7	10	66.7	

Calculated by Chi-Square test, unless otherwise indicated.

a Calculated by Kruskal-Wallis test.

**TABLE 3:** Univariate and multivariate analysis of factors associated with TTR according to the Cox Proportional Hazards Model.

Variable	Univariate			Multivariate		
	HR	95%	*P*	HR	95%	*P*
Age, years (≥58 *vs* < 58)	0.861	0.440−1.688	0.664			
Gender	0.753	0.392−1.447	0.395			
Tumor size	3.286	1.505−7.176	0.003	3.413	1.116−5.365	0.001
Tumor location	0.817	0.262−2.547	0.728			
Histological subtypes	1.032	0.453−2.351	0.940			
Differentiation	1.025	0.388−2.709	0.960			
Perineural invasion	1.840	0.929−3.645	0.080			
Vascular invasion	2.833	1.181−6.798	0.020	3.315	1.534−7.165	0.002
T stage	1.284	0.578−2.855	0.539			
*N* stage	1.739	0.555−5.446	0.342			
Distant metastasis	1.465	0.605−3.546	0.397			
TNM stage	0.785	0.182−3.381	0.745			
MRS	1.015	1.005−1.024	0.002	1.012	1.003−1.020	0.007

*p* < 0.05 represents statistical significance; HR, hazard ratio; CI, confidence interval.

### MRS-Based Nomogram Construction

**FIGURE 3:**
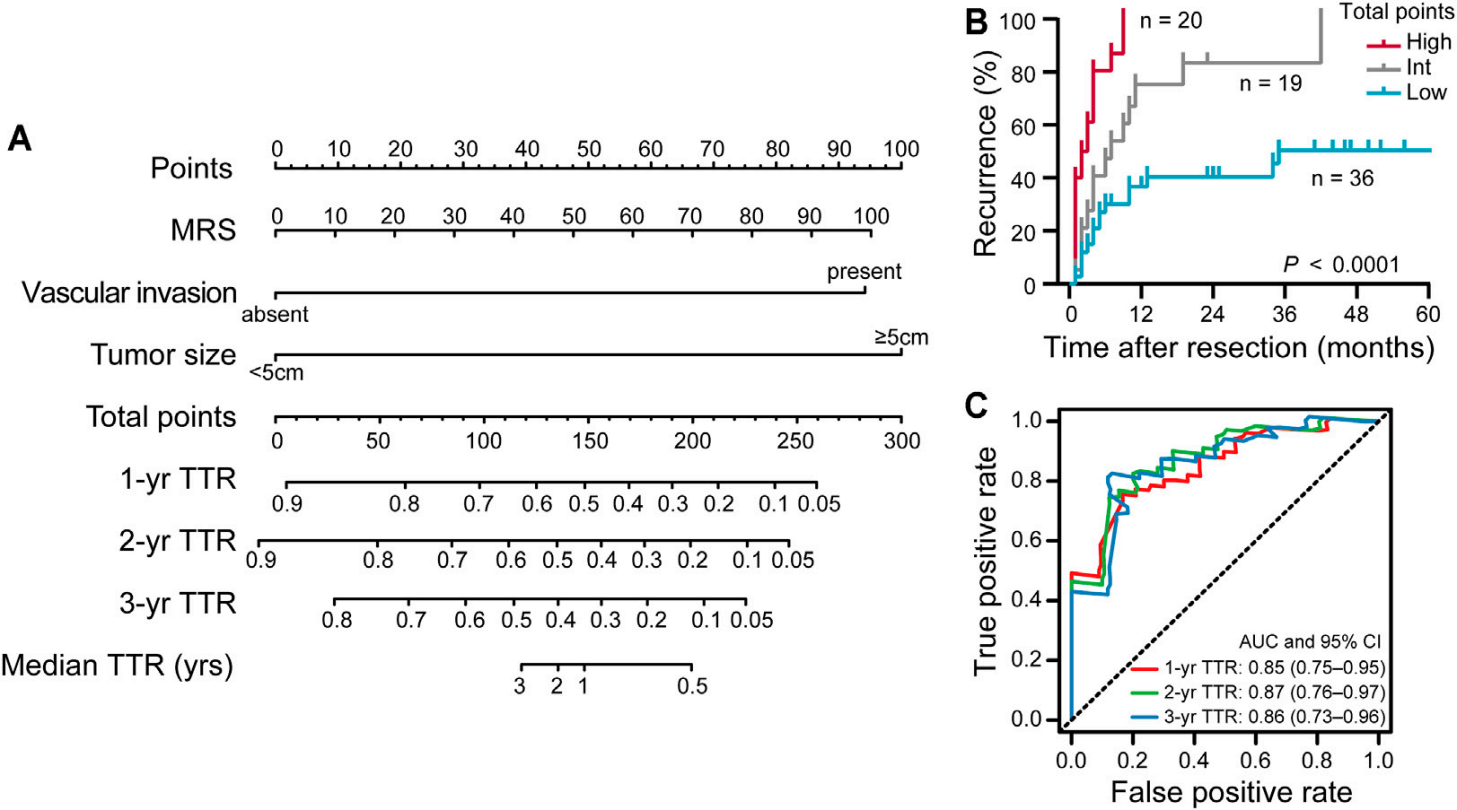
The construction and prognostic performance of the MRS-based nomogram. **(A)** The MRS-based nomogram for predicting time to recurrence of iCCA after resection. **(B)** Kaplan-Meier survival analysis of TTR according to the total points given by the nomogram. Patients were divided into three subgroups (Low: 0–191.3; Int: 191.3–328.4; High: 238.4–300) based on cutoff scores generated by X-tile program (Supplementary Figure S2). **(C)** ROC curve analysis showed the discriminatory ability of MRS-based nomogram for predicting 1, 2, and 3 years recurrence of iCCA after resection. The area under curve (AUC) was shown as the mean and 95%CI.

To exemplify the utility of the MRS in predicting iCCA patients’ risk of 1-, 2-, and 3-years recurrence after resection surgery, we conduct a nomogram (on a scale of 0–300), which incorporated abovementioned three independent prognostic factors (Table 3), namely, the MRS (as a continuous variable), the tumor size and the status of vascular invasion. The nomogram showed that the tumor size and the MRS contributed significantly to the prognosis, followed by the status of vascular invasion (Figure 3A). ROC curve analysis showed that the MRS-based nomogram had notable discriminatory abilities in predicting recurrence. A higher nomogram score suggest a greater risk of postoperative recurrence (Figure 3B). The AUCs of the nomogram predicting 1-, 2-, and 3-year recurrence were 0.85 (95% CI, 0.75–0.95), 0.87 (95% CI, 0.76–0.97) and 0.86 (95% CI, 0.73–0.96), respectively (Figure 3C).

### Validation and Clinical Usefulness of the MRS-Based Nomogram

**FIGURE 4:**
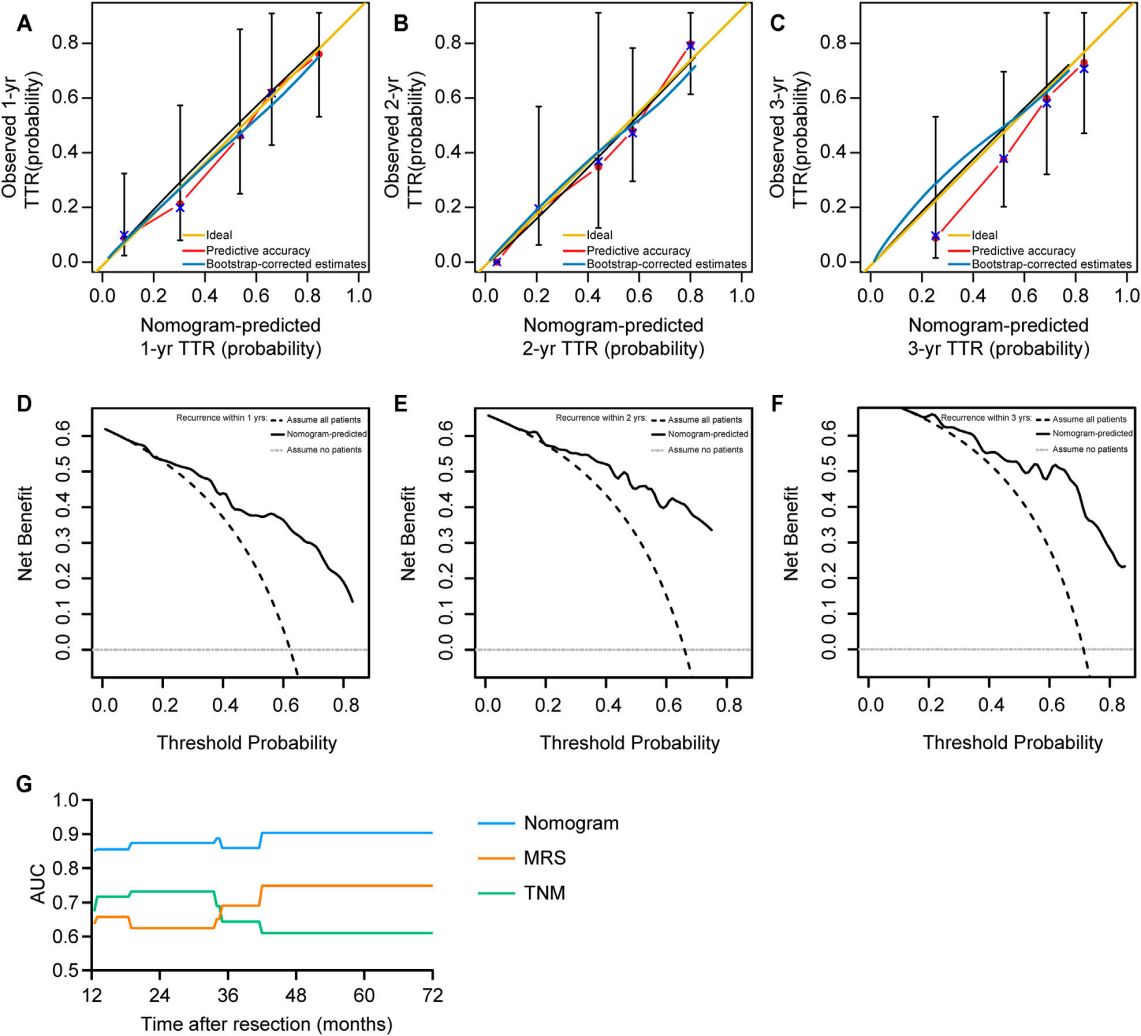
The calibration and clinical usefulness of the MRS-based nomogram. **(A–C)** The calibration curves for predicting iCCA patients’ postoperative 1, 2, and 3 years recurrence. **(D–F)** Decision curve analysis (DCA) of the clinical usefulness for this TTR nomogram. **(G)** The AUC values of the ROC curve analysis for the MRS-based nomogram at different time points was compared with that of the MRS alone or the TNM staging system by time-dependent ROC curves.

We validated this MRS-based nomogram model for Somers’ *D*_*xy*_ rank correlation between predicted log hazard and observed survival time, and for slope shrinkage. The bootstrap is used (with 300 resamples) to penalize for possible overfitting. The corrected estimate of how well the model will discriminate prognosis in the future is *D*_*xy*_ = 0.48, which is equivalent to a C-index of 0.74 and is very close to the apparent *D*_*xy*_ = 0.50 (Supplementary Table S2). The slope shrinkage factor is 0.93. These data consistently suggested a low level of overfitting. Next, bootstrap overfitting-corrected calibration curves are estimated. The calibration analysis demonstrated good agreement between the predicted and ideal accuracy, indicated that the MRS-based nomograms have good stability and reliability for predicting 1, 2, and 3 years recurrence (Figures 4A–C).

To analyze the clinical usefulness, the DCA was used. The results showed that the MRS-based nomogram demonstrated the net benefit of nomogram-assisted decisions at a wide range of threshold probability (Figures 4D–F). Finally, the discriminative capacity of the MRS-based nomogram was compared with that of the MRS alone or the TNM staging classification. Time-dependent ROC curve revealed that the MRS-based nomogram had superior performance than MRS alone and the TNM staging system (Figure 4G). These data indicate the high discrimination ability of the MRS-based nomogram for recurrence of iCCA patients after resection surgery.

## Discussion

Accurate measurement of the recurrence risk of tumor plays an essential role in effective postoperative management and physician-patient communication, particularly for iCCA, which is characterized by a high recurrence rate after surgery (Mavros et al., 2014). In this study, we verified the prognostic value of MRS (based on the *in situ* IHC features of CD11b and CD169) and built a predictive nomogram in iCCA which exhibited robust prognostic power for postoperative recurrence and represented a good auxiliary for clinical decisions. These findings indicate the clinical potential of MRS in iCCA, in addition to HCC.

As innate immune cells, myeloid cells are the first-line defenders against environmental threats. These cells are also important components of the tumor microenvironment that have complex interplays with the neoplastic and other stromal cells. Different subtypes of myeloid cells can co-exist in the tumor microenvironment, playing synergistic, opposing or non-interfering roles in the progression of cancer and in the response to therapeutic treatments (Ochoa et al., 2007; Ahn et al., 2010; Chen et al., 2013; Xu et al., 2016; Sabbatino et al., 2018). In our recent study (Wu, et al., 2020), we screened nine myeloid markers in both intra-tumoral and peri-tumoral tissues of HCC and then built a myeloid signature (named MRS) based on the IHC features of CD11b and CD169 in the intra-tumoral region. CD11b is a common myeloid marker widely expressed on myeloid cells. Previous studies reported the correlation of CD11b and poor clinical outcomes in some type of cancers, as well as its therapeutic potential (Ahn et al., 2010; Chen et al., 2013; Ochoa et al., 2007; Sabbatino et al., 2018; Xu et al., 2016). CD169 is often viewed as a macrophage marker in most cancers. The abundance of CD169^+^ macrophages in solid tumors is inconsistently correlated with the prognosis of the patients, indicating the heterogeneity and plasticity of CD169^+^ macrophages in different organs (Asano et al., 2018; Ohnishi et al., 2013; Ohnishi et al., 2016; Stromvall et al., 2017). The CD11b-CD169-based MRS showed remarkable discriminatory power for predicting the TTR and OS of HCC patients after surgical resection (Wu et al., 2020). Although HCC and iCCA share similar tissue microenvironment, the *in situ* expression and the prognostic values of CD11b and CD169 had not been determined in iCCA. In this study, we examined the protein expression of CD11b and CD169 in 75 iCCA tissues by IHC. Similar to that in HCC, our results showed that the abundance of infiltrating CD11b^+^ cells in tissues was associated with the risk of postoperative recurrence in iCCA. CD169 alone, however, had no significant relationship with the postoperative prognosis in iCCA. Nevertheless, the MRS showed good discriminatory ability and was tested as an independent risk factors for recurrence of iCCA. It is interesting that, unlike that in HCC, the MRS was not able to predict the OS of iCCA patients. This is possibly due to the limited sample size and the different postoperative/postrecurrence treatments for the patients. In conclusion, we verified that the MRS may serve as a valuable prognostic scoring system for predicting postoperative recurrence risk for iCCA patients.

Currently, the TNM staging system is most widely used for prognostic purpose in iCCA. Nevertheless, the 8th edition of TNM staging system, as well as other traditional staging systems, is mainly based on the anatomic factors and stratifies patients with OS, but lacks the ability to predict time to recurrence (Okabayashi et al., 2001; Nathan et al., 2009; Sakamoto et al., 2016; Lee and Chun 2018). Nomograms are statistical models developed to maximize predictive accuracy and this tool has shown its advantages in personalized prognosis in various cancers (Cheng et al., 2019). Yet, most of nomograms established to date for iCCA patients mainly focused on clinicopathologic characteristics (Jing et al., 2018; Liu et al., 2018; Zhang et al., 2018; Zhang et al., 2019; Chen et al., 2020; Sun et al., 2020; Yu et al., 2020). In this study, we built a nomogram based on the MRS, a signature of the myeloid response balance in the tumor immune microenvironment, to provide personalized prognosis for patients with iCCA after resection. In addition to MRS, this nomogram only included two other independent clinicopathologic risk factors, namely the tumor size and the status of vascular invasion, to predict early recurrence. The number of variables in this TTR nomogram were not only less than the nomogram we constructed in HCC (Wu et al., 2020), but also less than the majority of reported prognostic nomograms in iCCA (Burkhart and Pawlik 2017; Cheng et al., 2019). Therefore, this MRS-based nomogram in iCCA is rather simple and readily adaptable for clinical application. In comparison, this nomogram also showed robust discriminatory abilities and considerable stability in predicting recurrence in patients with iCCA after hepatectomy, which was superior to the MRS itself and the current TNM staging systems. The bootstrap corrected Somers’ *Dxy* rank correlation coefficient is 0.4795 (Supplementary Table S2), which is equivalent to a C-index of 0.7398. These results indicate that this MRS-based nomogram is a simple, useful and reliable prognostic tool in iCCA.

Although we have developed an improved prognostic tool to predict the postoperative recurrence of iCCA, there are some limitations in this study. First, this is a retrospective design with relatively small samples (due to the low incidence of iCCA, only accounting for 5–10% of all primary liver cancers). Second, this is a single-center study lack of external validation. A larger cohort of iCCA samples and a multicenter validation are warranted in further study. In addition, irrespective of its potential importance in the prognostic of iCCA, the MRS or this nomogram does not specifically make treatment recommendations. More research is needed to shed light on therapeutic value of the MRS in iCCA, like in HCC (Wu et al., 2020).

In conclusion, in this study we investigated the *in situ* expression of CD11 and CD169 in iCCA. The prognostic value of the CD11b-CD169-based MRS was not only shown in predicting the postoperative recurrence of iCCA, but was also applied into a prognostic nomogram that may help the personalized prediction and clinical decision. Together, these data support the valuable potential of myeloid signatures in oncology and suggest that assessing the myeloid response balance in the tumor immune microenvironment can provide useful information for precise prognostic prediction in cancers.

## Data Availability

The original contributions presented in the study are included in the article/Supplementary Material, further inquiries can be directed to the corresponding authors.

## Abbreviations

AJCC, American Joint Committee on Cancer; AUC, area under curve; DCA, decision curve analysis; HCC, hepatocellular carcinoma; iCCA, intrahepatic cholangiocarcinoma; IHC, immunohistochemistry; MRS, myeloid response score; OS, Overall survival; ROC, receiver operating characteristic; TTR, the time to recurrence.
