# Supplementary material for: A Myeloid Signature-Based Nomogram Predicts the Postoperative Recurrence of Intrahepatic Cholangiocarcinoma
Source: Front Mol Biosci. 2021 Oct 13;8:742953. doi: 10.3389/fmolb.2021.742953 (PMC8548627; doi:10.3389/fmolb.2021.742953)
Supplement: Supplementary file 1 [file DataSheet1.pdf]

Supplementary Materials for

A Myeloid Signature-based Nomogram Predicts the Postoperative Recurrence of Intrahepatic Cholangiocarcinoma

Jing Liang[†], Hui Zhou[†], Xiang-Qi Huang[†], Yan-Fei Liu, Lei Zhang, Dan He, Yongmei Cui, Jinrui Guo, Kunpeng Hu[*], Chong Wu[*]

*** Correspondence to:**

Kunpeng Hu

hukpeng@mail.sysu.edu.cn

Chong Wu

Wuchong5@mail.sysu.edu.cn

[†]These authors have contributed equally to this work

This file includes:

Supplementary Figure 1. The optimum cutoff of MRS determined by X-tile plot.

Supplementary Figure 2. The optimum cutoff of MRS-based nomogram determined by X-tile plot.

Supplementary Table 1. Univariate and multivariate analysis of factors associated with OS according to the Cox Proportional Hazards Model (MRS as a continuous variable).

Supplementary Table 2. Bootstrap validation and bias-corrected estimates of the MRS-based nomogram for predicting postoperative recurrence of iCCA.

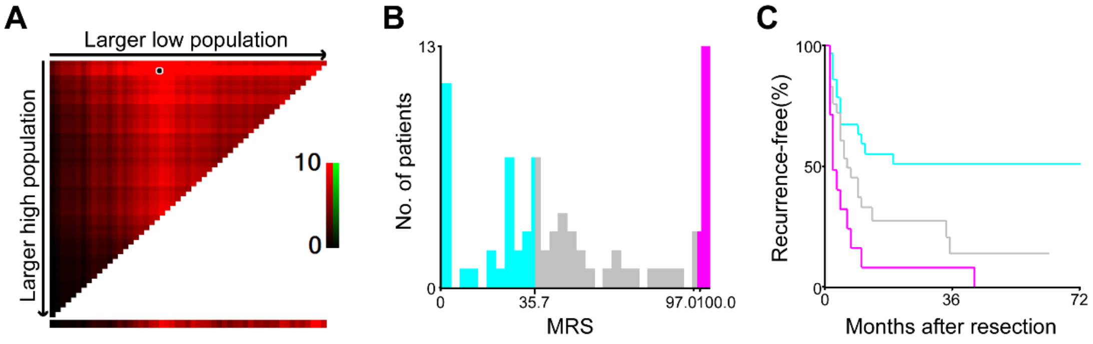

Supplementary Figure 1. The optimum cutoff of MRS determined by X-tile plot. (A) The optimum cutoff point (highlighted by the black/white circle) of MRS grouping was determined by X-tile plot. (B) Distribution of patients with different MRS, the cutoff point was shown on the X axis. (C) Kaplan-Meier plot showed the distinct clinical outcomes of patients with different MRS.

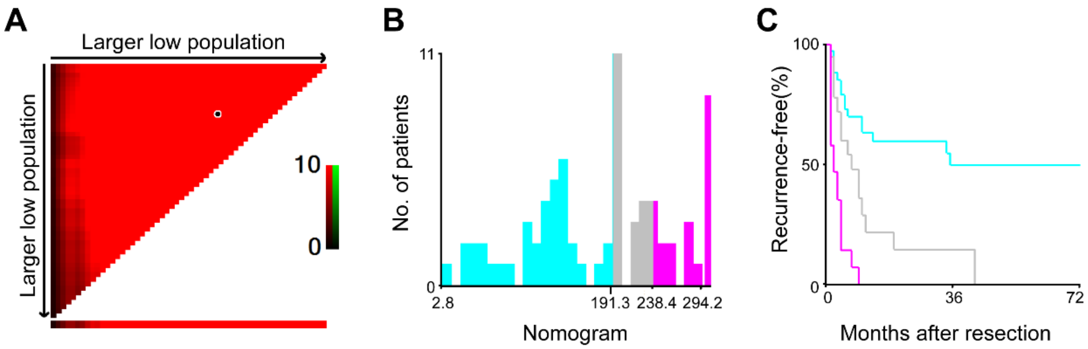

Supplementary Figure 2. The optimum cutoff of MRS-based nomogram determined by X-tile plot. (A) The optimum cutoff point (highlighted by the black/white circle) of MRS-based nomogram grouping was determined by X-tile plot. (B) Distribution of patients with different nomogram scores, the cutoff point was shown on the X axis. (C) Kaplan-Meier plot showed the distinct clinical outcomes of patients with different nomogram scores.

Supplementary Table 1. Univariate and multivariate analysis of factors associated with OS according to the Cox Proportional Hazards Model (MRS as a continuous variable)

Variable	Univariate			Multivariate		
	HR	95% CI	*P*	HR	95% CI	*P*
Age, years (≥ 58 *vs* <58)	2.825	1.269-6.287	0.011	1.669	0.883-3.156	0.115
Gender	1.214	0.595-2.476	0.594			
Tumor size	2.731	1.121-6.655	0.027	2.432	1.108-5.339	0.027
Tumor location	0.384	0.102-1.441	0.156			
Histological subtypes	0.508	0.218-1.186	0.117			
Differentiation	1.625	0.665-3.972	0.287			
Perineural invasion	0.715	0.329-1.555	0.397			
Vascular invasion	2.097	0.877-5.015	0.096			
T stage	2.737	1.089-6.877	0.032	1.519	0.761-3.029	0.236
N stage	2.848	0.705-11.507	0.142			
Distant metastasis	1.543	0.614-3.875	0.356			
TNM stage	0.348	0.060-2.026	0.240			
MRS	1.001	0.991-1.012	0.783			

NOTE. $P < 0.05$ represents statistical significance; HR, hazard ratio; CI, confidence interval.

Supplementary Table 2. Bootstrap validation and bias-corrected estimates of the MRS-based nomogram for predicting postoperative recurrence of iCCA

Index	Original sample	Training sample	Test sample	Optimism	Corrected index	*n*
D_{xy}	0.4987	0.4940	0.4747	0.0193	0.4795	300
R^2	0.3658	0.3737	0.3443	0.0294	0.3363	300
Slope	1.0000	1.0000	0.9332	0.0668	0.9332	300
D	0.0938	0.0990	0.0870	0.0120	0.0818	300
U	-0.0057	-0.0058	0.0029	-0.0087	0.0030	300
Q	0.0996	0.1048	0.0840	0.0208	0.0788	300

NOTE. “Training” refers to accuracy when evaluated on the bootstrap sample used to fit the model, and “Test” refers to the accuracy when this model is applied without modification to the original sample. D_{xy}: Somers’ rank correlation between predicted probability and observed incidence; R^2: the fraction of log likelihood explained by the model of the log likelihood that could be explained by a perfect model, penalized for the complexity of the model; Slope, calibration slope, which is the slope of predicted log odds *vs.* true log odds; *D*: discrimination index, which is the −2 log likelihood at the shrunken linear predictor, penalized for estimating one parameter; *U*: unreliability index, which measures how far the model maximum log likelihood (which allows for an overall slope correction) is from the log likelihood evaluated at “frozen” regression coefficients. *Q*: logarithmic accuracy score, which is a normalized and penalized −2 log likelihood that is evaluated at the uncorrected linear predictor.
